# Supplementary figures and images for: Predicting PY motif-mediated protein-protein interactions in the Nedd4 family of ubiquitin ligases
Source: PLoS One. 2021 Oct 12;16(10):e0258315. doi: 10.1371/journal.pone.0258315 (PMC8509885; doi:10.1371/journal.pone.0258315)

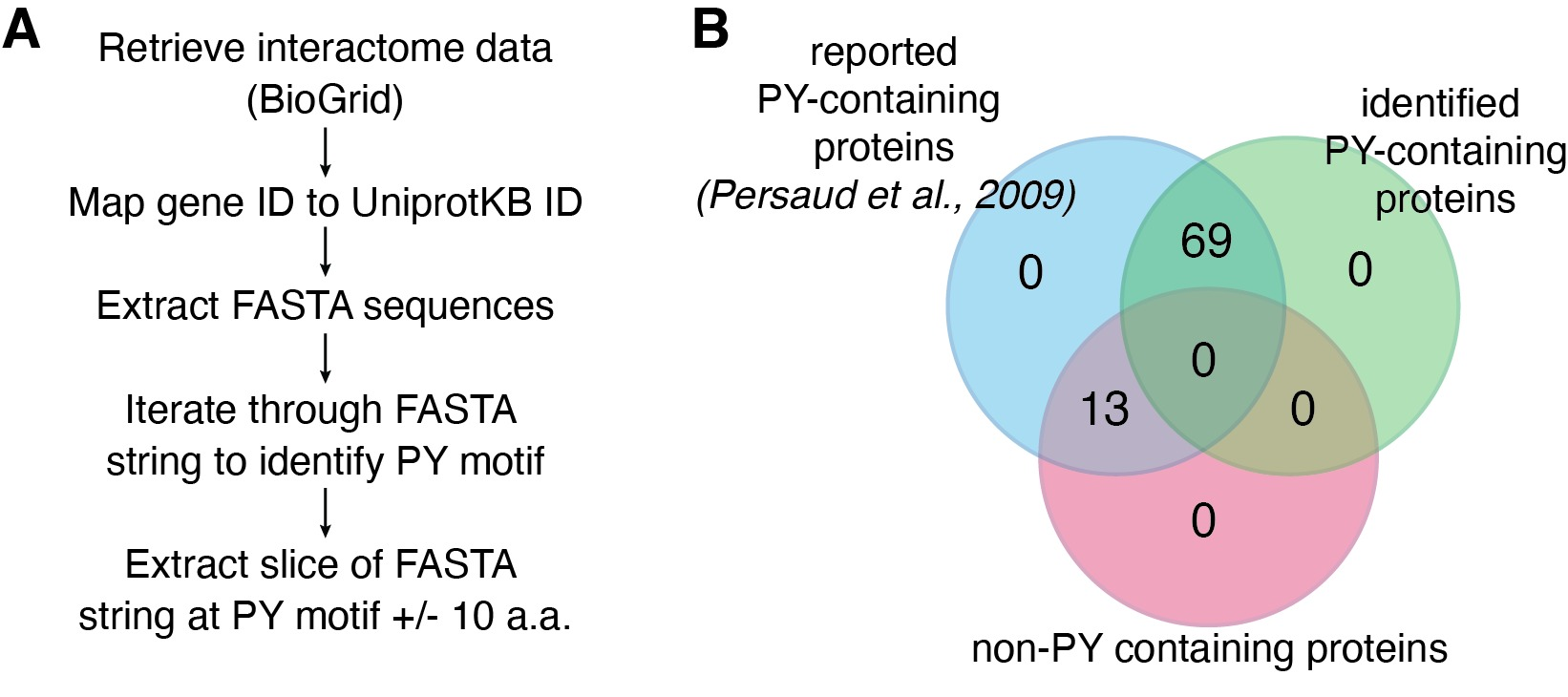

Supplement: S1 Fig — (A) The workflow of PxYFinder implements a python-based script to rapidly identify PY motifs from protein sequences as FASTA format. Protein interaction datasets can be retrieved from public databases such as BioGrid. PxYFinder script allows conversion from interaction list to UniProt ID for FASTA accession. FASTA sequences are then processed as data strings for identification of PY motif and extraction of PY-containing regions. (B) Validation of PxYFinder script with manual confirmation against a previously published dataset34 of PY motif-containing proteins reveals errors in previously identified PY motifs. (TIF) [file pone.0258315.s001.tif]

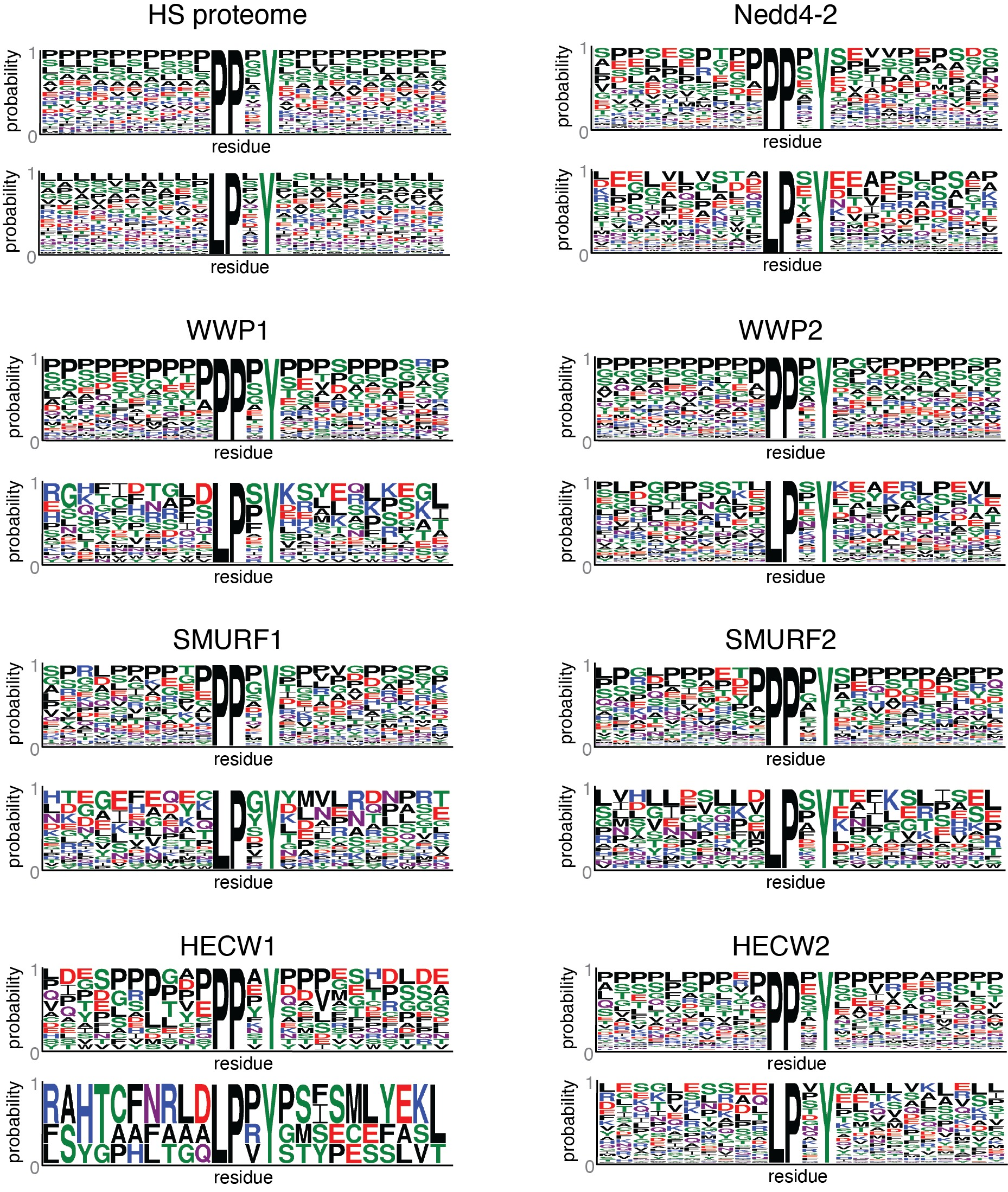

Supplement: S2 Fig — Sequence logo diagrams were used to identify consensus sequences in PY motifs and in surrounding regions (± 10 amino acids) for Nedd4 family members and for all Homo Sapiens proteins that have SwissProt annotation available in the UniProt database (labeled as HS proteome). Sequence logos for Nedd4-1 and ITCH are excluded from this figure as they are presented as representative images in Fig 2. Sequence logo analysis reveals that PPxY motifs are more likely to occur in proline-rich regions than LPxY motifs, and amino acid identity at the x position is more conserved in PPxY motifs across the Nedd4 family and proteome than in LPxY motifs. (TIF) [file pone.0258315.s002.tif]

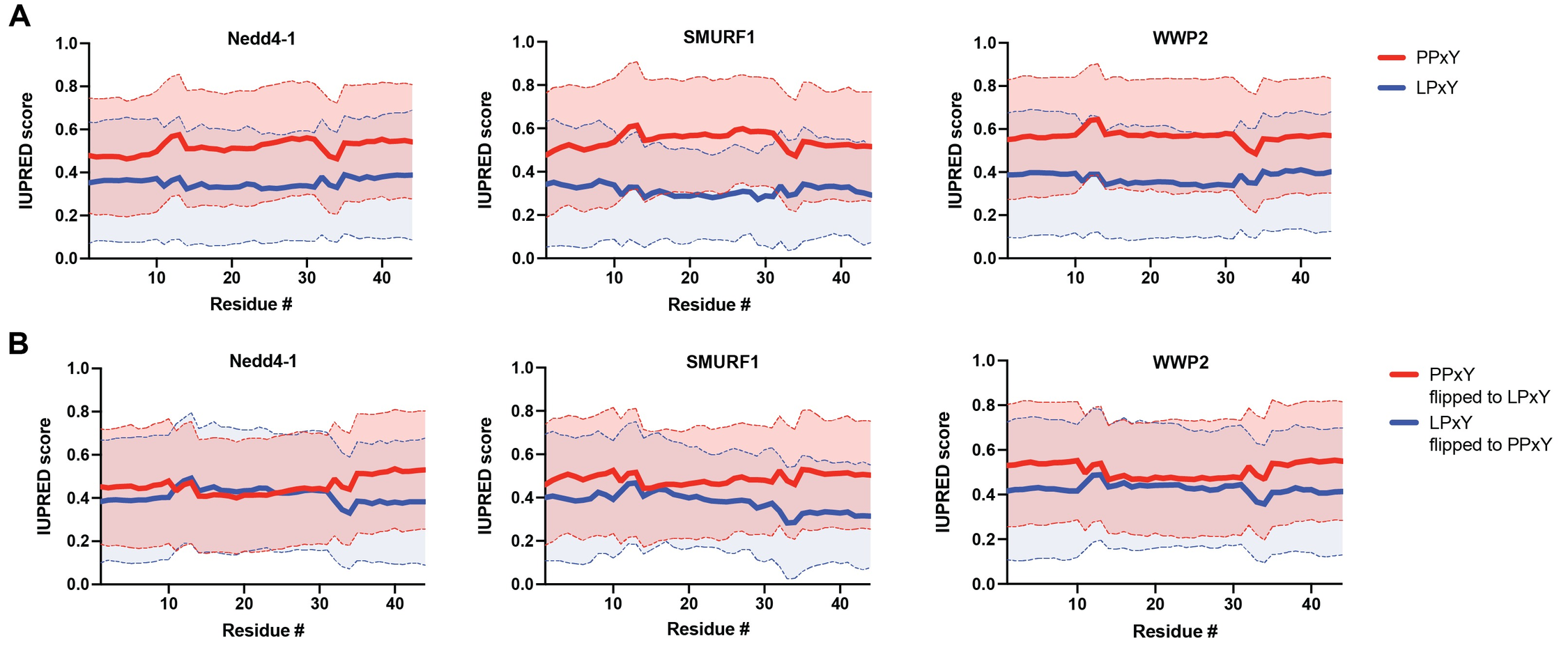

Supplement: S3 Fig — (A) As a first analysis, the predicted order of each PY motif containing Nedd4-family interactome member was analyzed using IUPred2A and disorder scores were extracted ± 20 amino acids surrounding the PY motif sequence. Nedd4-1, SMURF1, and WWP2 show similar trends in predicted order around the PY motifs, with PPxY motifs occurring in more disordered regions relative to LPxY. (B) PY motifs in each interactor were computationally flipped wherein PPxY was substituted for LPxY and vice versa. Interactors were then re-analyzed with IUPred2A, revealing that substitution of P for L in the PY motif decreased predicted disorder values in PPxY-containing proteins while substation of L for P increased predicted disorder. This trend was consistent for all three interactomes analyzed. (TIF) [file pone.0258315.s003.tif]

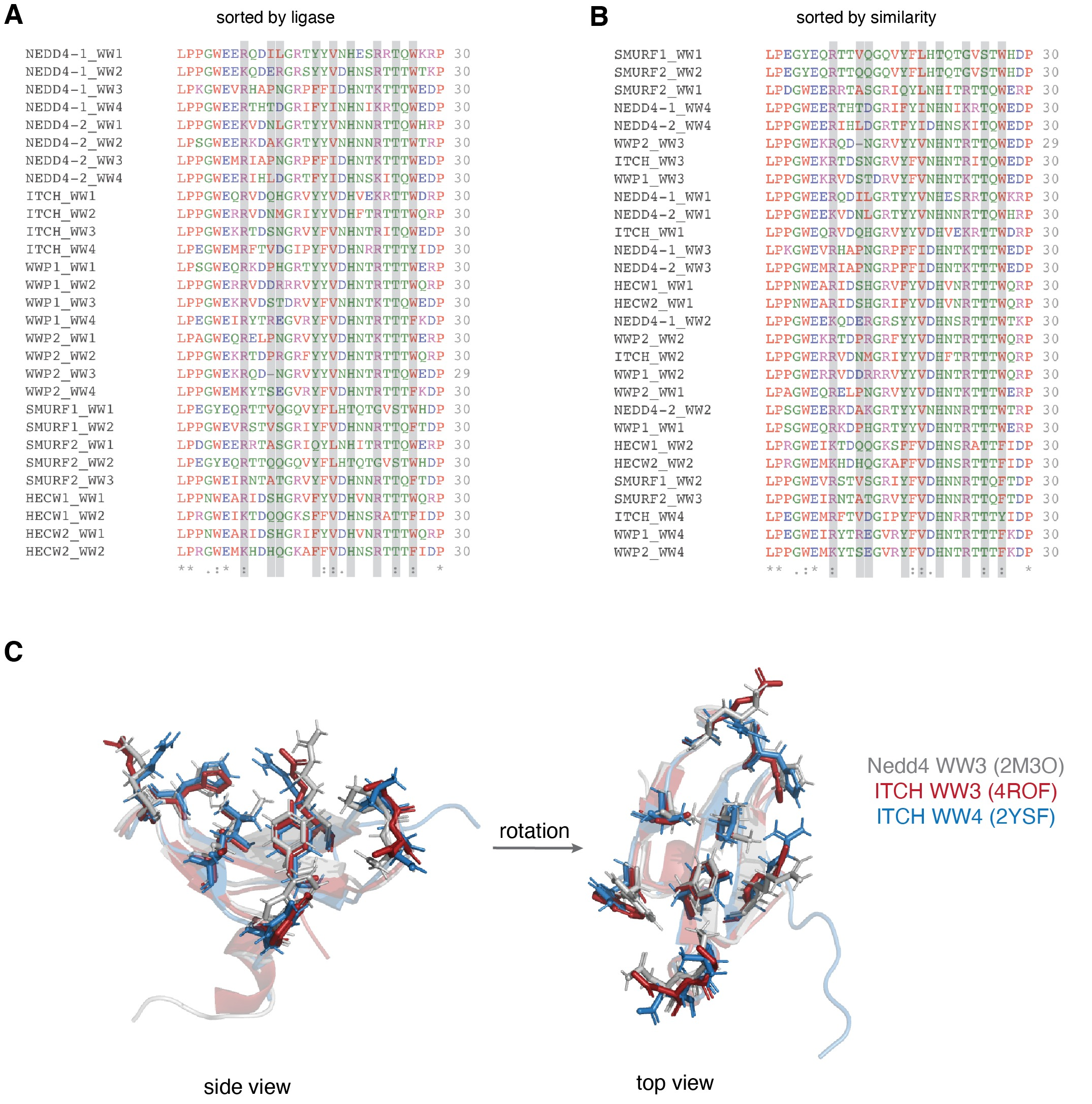

Supplement: S4 Fig — Sequence alignments of WW domains from Nedd4 family members sorted by (A) ligase and (B) similarity show moderate sequence conservation, with high conservation of key residues in the binding interface (highlighted in grey). (C) Alignment of three WW domain structures with varying sequence similarity show high conservation of structure and of positioning of key residues despite differences in residue identity. (TIF) [file pone.0258315.s004.tif]

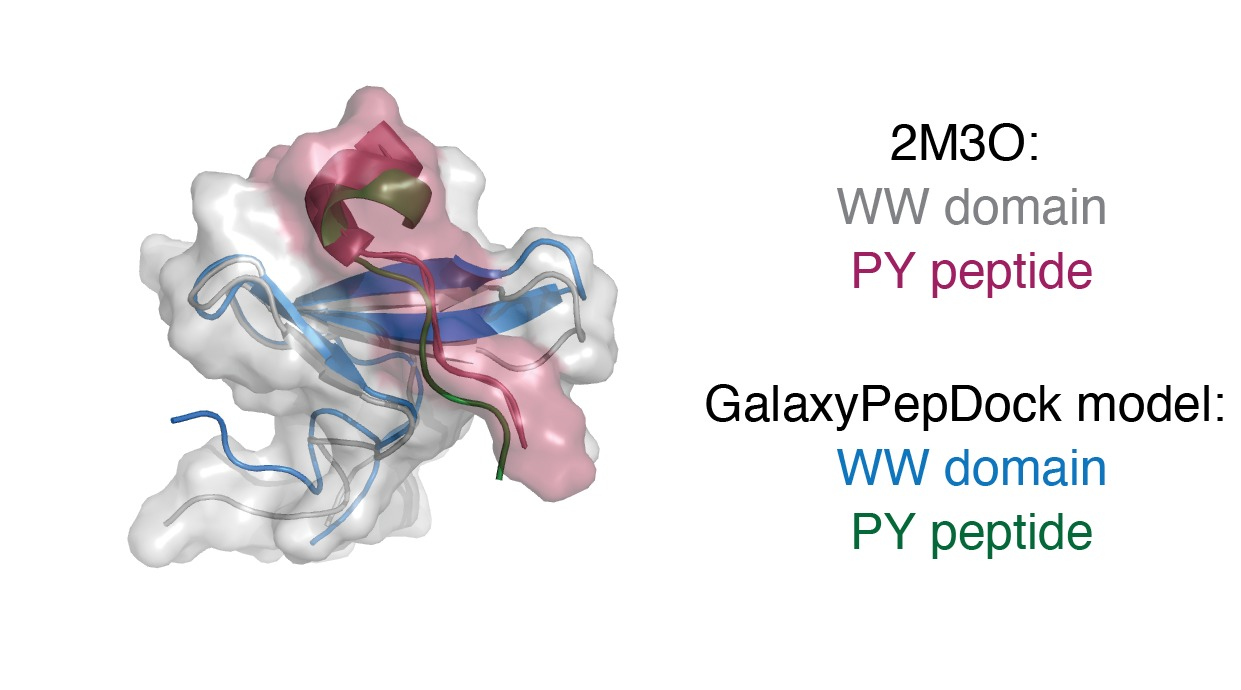

Supplement: S5 Fig — As a test of GalaxyPepDock template-based docking reliability, the native peptide substrate of Nedd4 WW domain (reported in PDB structure 2M3O) was docked to the apo-WW domain, extracted from PDB 2M3O. Alignment of the native complex (2M3O, peptide shown in red; WW domain in grey) with the docked complex (via GalaxyPepDock; peptide in green; WW domain in blue) show reliable docking of the peptide with retention of conformation and peptide-WW domain contacts. (TIF) [file pone.0258315.s005.tif]

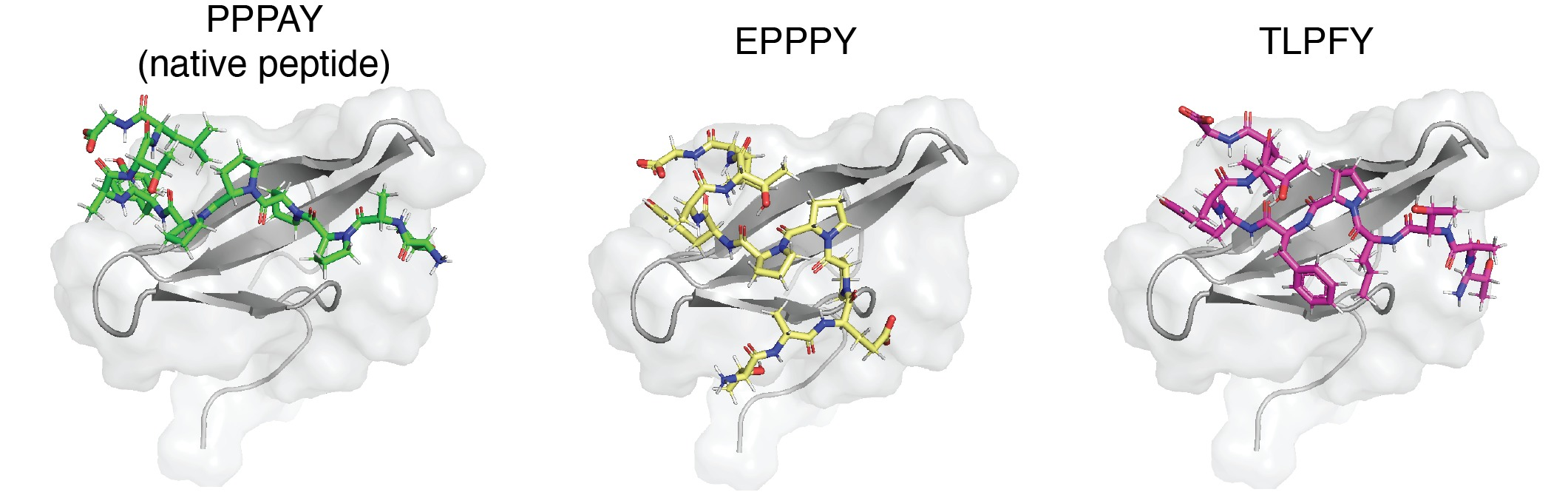

Supplement: S6 Fig — A sampling of peptide conformations from computational docking of the rationally designed peptide library demonstrates the variety of intramolecular contacts that the PY peptides can form with the WW domain structure. Binding energies of the representative peptides shown here are presented in Fig 4. (TIF) [file pone.0258315.s006.tif]

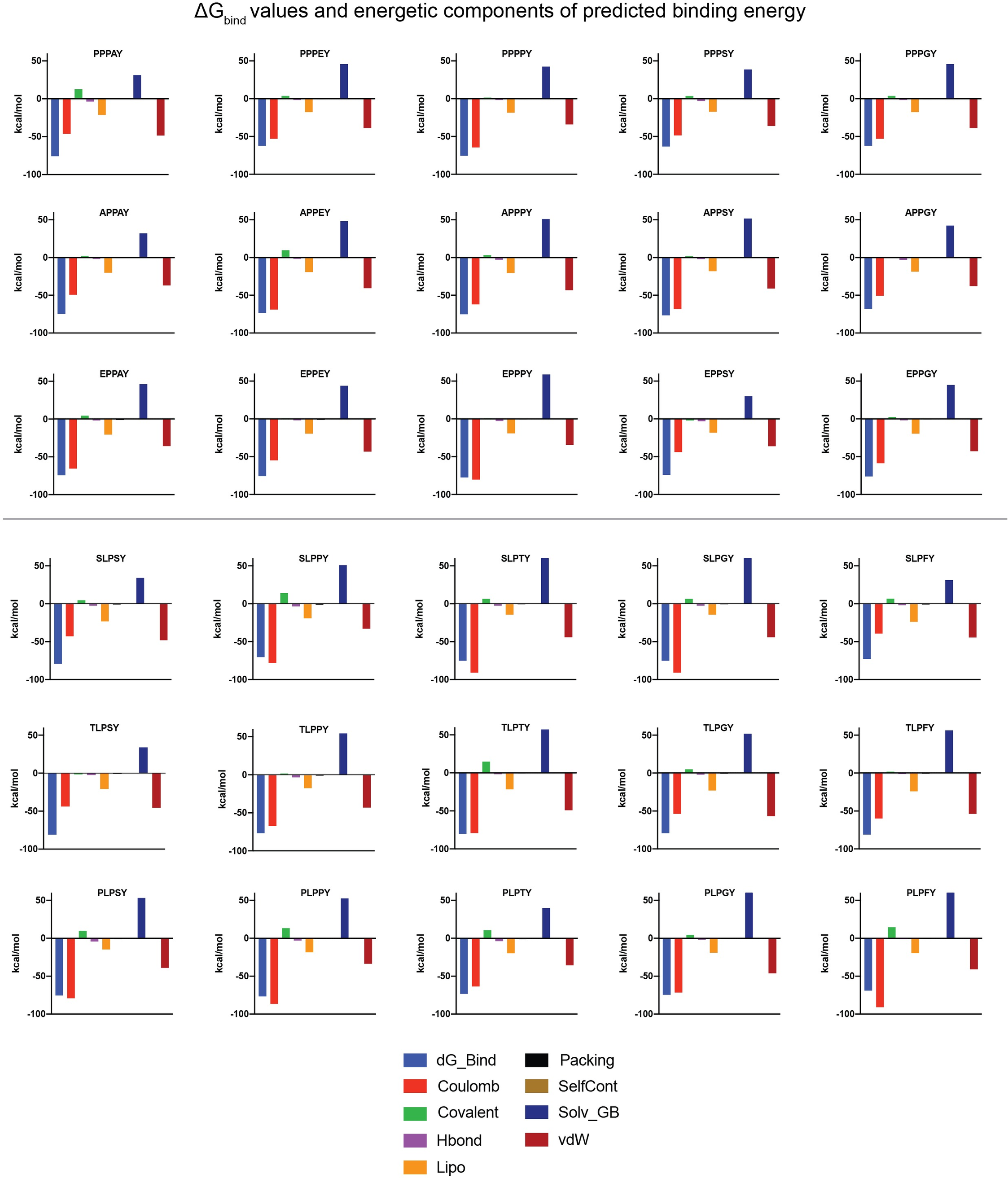

Supplement: S7 Fig — ΔGbinding and energetic components that contribute to ΔGbinding are shown here as calculated with the Schrodinger Prime MM-GBSA tool. Energies are given in kcal/mol, and energy contributions are shown for all 30 members of the rationally designed PY peptide library. (TIF) [file pone.0258315.s007.tif]

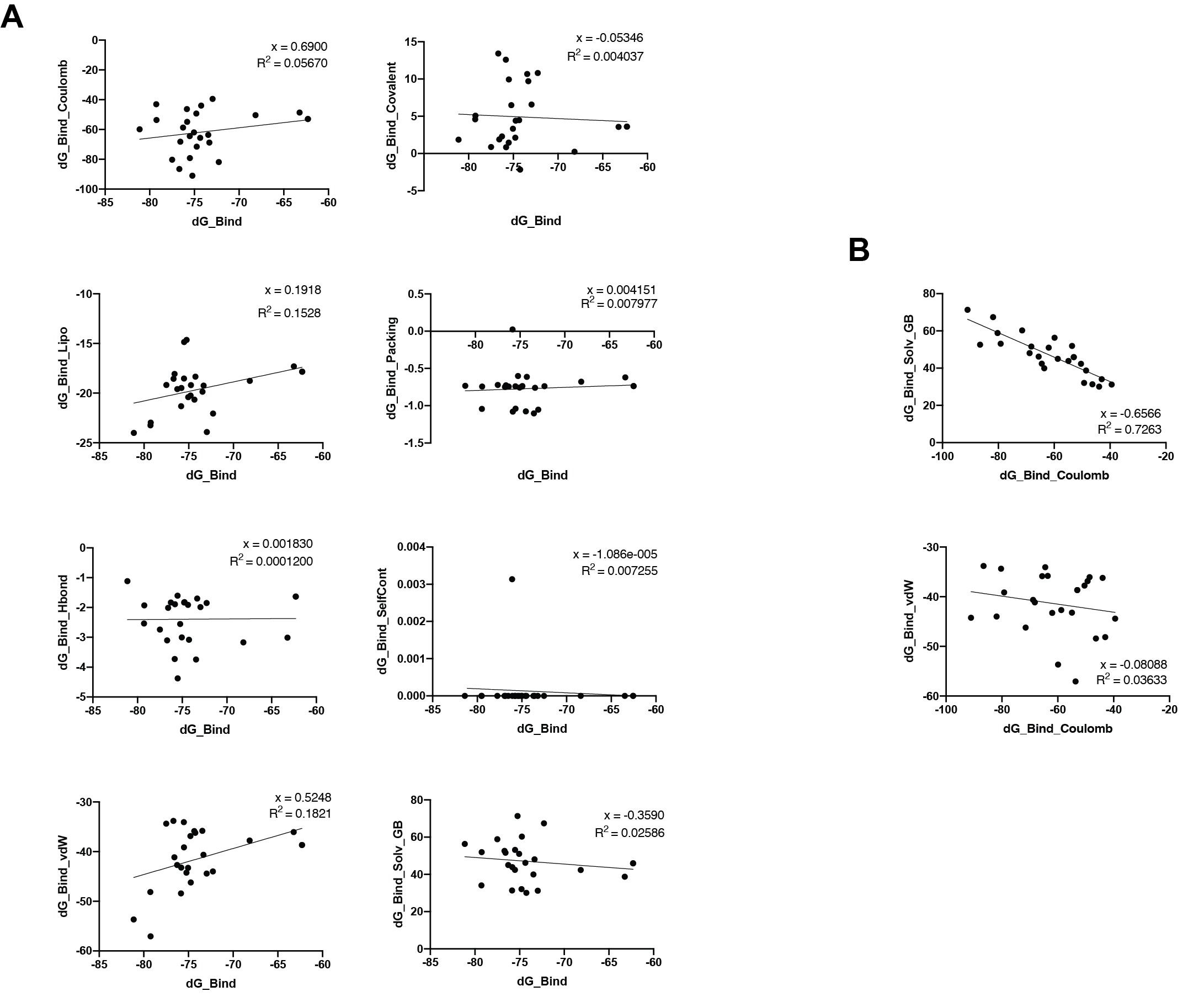

Supplement: S8 Fig — Correlation of calculated energies (ΔGbinding and ΔGbinding sub-components) across the peptide library show that (A) some energetic contributions are more strongly correlated to overall binding (ΔGbinding) relative to other components. (B) Correlation of solvation (Solv_GB) and van der Waals (vdW) components of ΔGbinding with coulombic interactions shows that solvation is more strongly correlated with coulombic interactions than van der Waals interactions. Specifically, stronger (more negative) coulombic interactions correlate with more positive solvation energies. Values calculated with Schrodinger Prime MM-GBSA and presented in kcal/mol. Simple linear regression analysis and data visualization performed in Prism GraphPad. X = slope of linear regression line of best fit; R2 provided as measure of goodness of fit. (TIF) [file pone.0258315.s008.tif]

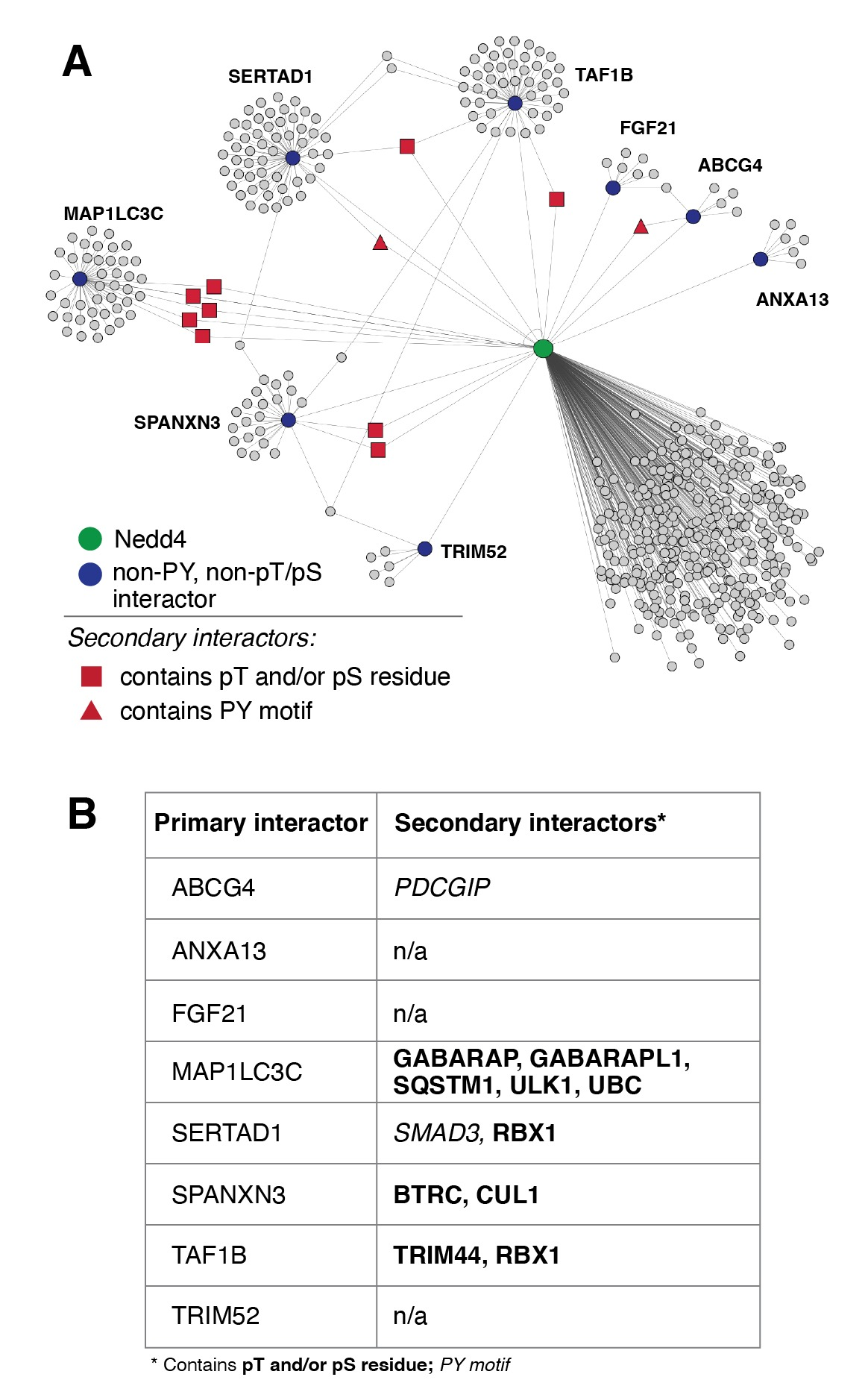

Supplement: S9 Fig — (A) Interaction networks of Nedd4-1 (green node) and non-PY, non-pT/pS substrates of Nedd4 (blue nodes) were retrieved from BioGrid and merged using Cytoscape, revealing secondary interactors that are functionally related and contain either PY (red triangles) or pT and/or pS residues (red squares). (B) Identity of primary and secondary interactors depicted in A are presented where bolded proteins contain pT and/or pS residues while italicized proteins contain PY motifs. (TIF) [file pone.0258315.s009.tif]
