# Supplementary material for: Predicting PY motif-mediated protein-protein interactions in the Nedd4 family of ubiquitin ligases
Source: PLoS One. 2021 Oct 12;16(10):e0258315. doi: 10.1371/journal.pone.0258315 (PMC8509885; doi:10.1371/journal.pone.0258315)
Supplement: S1 Table — (DOCX) [file pone.0258315.s010.docx]

**Table S1: PY-containing proteins correctly identified from test set using PxYFinder.**

| **UniProt ID** | **Gene name** | **Reported PY motif (Persaud et al., 2009)**^34^ |
| --- | --- | --- |
| P00519 | ABL1 | PPFY |
| P42684 | ABL2 | PPFY |
| P16112 | ACAN | LPNY |
| P36896 | ACVR1B | LPYP |
| P31749 | AKT1 | LPFY |
| Q9Y243 | AKT3 | LPFY |
| Q01433 | AMPD2 | LPEY |
| O43315 | AQP9 | LPFY |
| Q9UQB8 | BAIAP2 | PPDY |
| Q8WY36 | BBX | LPQY |
| P36894 | BMPR1A | LPYY |
| Q9P1Z2 | CALCOCO1 | LPPY |
| Q8IU85 | CAMK1D | PPFY |
| Q16566 | CAMK4 | LPEY |
| Q00526 | CDK3 | LPDY |
| Q00535 | CDK5 | LPDY |
| O15247 | CLIC2 | PPRY |
| Q9NWM3 | CUEDC1 | PPCY, PPAY |
| Q9NUI1 | DECR2 | LPAY |
| Q08495 | DMTN | PPIY, LPAY |
| O95967 | EFEMP2 | PPGY |
| P55010 | EIF5 | PPTY |
| Q8TAM6 | ERMN | LPHY |
| P21802 | FGFR2 | LPQY |
| Q00688 | FKBP3 | PPKY |
| P17948 | FLT1 | PPDY |
| Q9P0K8 | FOXJ2 | PPLY |
| O14964 | HGS | PPEY |
| Q14721 | KCNB1 | LPPY |
| P43366 | MAGEB1 | PPRY |
| P11137 | MAP2 | PPSY |
| Q9Y2U5 | MAP3K2 | PPGY |
| Q99759 | MAP3K3 | PPGY |
| Q99683 | MAP3K5 | PPFY |
| P49137 | MAPKAPK2 | PPFY |
| Q16644 | MAPKAPK3 | PPFY |
| Q8IW41 | MAPKAPK5 | PPFY |
| Q7KZI7 | MARK2 | LPDY |
| P33993 | MCM7 | LPQY |
| P49406 | MRPL19 | LPEY |
| Q9NV92 | NDFIP2 | PPPY |
| P04629 | NTRK1 | PPVY |
| Q8N5Y8 | PARP16 | PPKY |
| Q8N4L2 | PIP4P2 | PPPY |
| Q9NRY6 | PLSCR3 | PPPY |
| P51817 | PRKX | PPVY |
| O14668 | PRRG1 | PPTY, PPEY |
| P51812 | RPS6KA3 | LPQY |
| Q9UBS0 | RPS6KB2 | LPPY |
| Q92541 | RTF1 | PPNY |
| Q96ER3 | SAAL | LPFY |
| O00141 | SGK1 | PPFY |
| Q9HBY8 | SGK2 | PPFY |
| Q9UGH3 | SLC23A2 | PPQY, LPIY |
| P84022 | SMAD3 | PPGY |
| Q8IWU6 | SULF1 | LPQY |
| Q15633 | TARBP2 | LPEY |
| Q9NUJ3 | TCP11L1 | PPAY |
| Q15562 | TEAD2 | LPGY |
| O43294 | TGFB1I1 | PPSY |
| P62072 | TIMM10 | PPHY |
| Q86TN4 | TRPT1 | LPKY |
| Q9BSL1 | UBAC1 | LPSY |
| O94941 | UBOX5 | PPVY |
| P45880 | VDAC2 | PPSY |
| Q969T9 | WBP2 | PPGY, PPPY, PPPY |
| P25490 | YY1 | PPDY |
| Q9NYG2 | ZDHHC3 | PPPY |
