# Supplementary material for: Predicting PY motif-mediated protein-protein interactions in the Nedd4 family of ubiquitin ligases
Source: PLoS One. 2021 Oct 12;16(10):e0258315. doi: 10.1371/journal.pone.0258315 (PMC8509885; doi:10.1371/journal.pone.0258315)
Supplement: S2 Table — (DOCX) [file pone.0258315.s011.docx]

**Table S2: Proteins identified as non-PY containing with PxYFinder but labeled as PY containing in test data set (from Persaud et al., 2009).^34^**

| **UniProt ID** | **Gene name** | **Reported PY motif**  **(Persaud et al., 2009)** | **PY motif identified with PxYFinder?** | **PY motif sequence identified manually** |
| --- | --- | --- | --- | --- |
| P32320 | CDA | LPLY | No | n/a |
| P11802 | CDK4 | LPDY | No | n/a |
| P49674 | CSNK1E | LPPY | No | n/a |
| Q8TDD1 | DDX54 | LPGY | No | n/a |
| P04062 | GBA | LPLY | No | n/a |
| O43448 | KCNAB3 | LPEY | No | n/a |
| Q9P015 | MRPL15 | LPEY | No | n/a |
| P16234 | PDGFRA | LPQY | No | n/a |
| P17252 | PRKCA | LPEY | No | n/a |
| Q6VN20 | RANBP10 | PPFY | No | n/a |
| P42680 | TEC | PPEY | No | n/a |
| Q6ZVM7 | TOM1L2 | PPPY | No | n/a |
| Q96A61 | TRIM52 | PPPY | No | n/a |
