# Supplementary material for: Predicting PY motif-mediated protein-protein interactions in the Nedd4 family of ubiquitin ligases
Source: PLoS One. 2021 Oct 12;16(10):e0258315. doi: 10.1371/journal.pone.0258315 (PMC8509885; doi:10.1371/journal.pone.0258315)
Supplement: S1 File — (ZIP) [file pone.0258315.s013.zip › PxYFinder/PxYFinder_UserGuide.pdf]

PxYFinder User Guide

Two programs are necessary in order to replicate the PY motif interactome analysis performed in "A bioinformatic and computational analysis of PY motif-mediated protein-protein interactions in the Nedd4 family of ubiquitin ligases".

"(1) Obtain GeneIds.py"

- This is a simple program which takes as input a BioGRID Tab 3.0 Format file for a protein (from thebiogrid.org) and outputs a .txt file containing a list of gene IDs of all the proteins shown to be interactors for the given protein of interest. This output file can be either uploaded to https://www.uniprot.org/uploadlists/ or the contents can simply be copy and pasted into the "Provide your identifiers" section of the page.
- In order to perform the analysis as described, there are only two variables that need to be manipulated within the program. Both are paths to specific files. The first, "BioGridPath", should be a string consisting of the path leading to the downloaded BioGRID Tab 3.0 file. The second, "GeneIDsOutput" should be a string containing the path leading to a new .txt file that will be created when the program runs (it doesn't need to exist beforehand).

"(2) PxYFinder.py"

- This program begins with the FASTA sequence file downloaded by mapping the output of the previous program onto UniProt. It consists of 5 sections and requires that 6 variables be manipulated in order to complete the analysis (one of which is a file path to the FASTA file downloaded from UniProt, and the rest of which are file paths to the files that will be created during the analysis).
    - **Section 1:** "FASTA TO CSV"; This section takes in the .fasta file from UniProt and outputs a .csv with columns for name, accession, and FASTA sequence for each interactor.
    - **Section 2:** "PY MOTIF ANALYSIS"; This section takes uses as input the .csv file created in Section 1 and outputs a new .csv file containing columns for name, accession, FASTA, PP, PPY, PPxY, and LPxY for each interactor (where the PP, PPY, PPxY, and LPxY columns contain either 0 or 1 indicating that the motif is not present, or the motif is present respectively).
    - **Section 3:** "SUMMARY OF MOTIFS (PERCENTAGE / BREAKDOWN)"; This section uses as input the .csv file created in Section 2, and outputs a .txt file containing a summary of the proportion of the interactome with each motif, also printing separate lists of interactors separated by whether or not a LPxY or PPxY motif is present.
    - **Section 4:** "OBTAIN SEQUENCES FOR WEBLOGO"; This section uses as input the .csv file created in Section 1, outputting a list of sequences containing either the PPxY motif or the LPxY and the 10 amino acids upstream and downstream each. This can be used with a Weblogo program to create a sequence logo.
    - **Section 5:** "OBTAIN ACCESSION NUMBERS FOR PANTHER"; This section uses as input the .csv file created in Section 2 and generates a .txt file containing the

UniProt accessions for all interactors separated by motif presence for use with PANTHER.

- Variables to be manipulated:
  - **FastaFilePath:** This variable is a string which consists of the file path of the .fasta file downloaded from UniProt after mapping the GeneIDs of generated by "*(1) Obtain GeneIds.py"* onto UniProt.
  - **csv_one_path:** This variable is a string which consists of the file path of the .csv file containing columns for name, accession, and FASTA sequence of the entire interactome. The file need not exist before the start of the analysis, but it should end in .csv.
  - **csv_two_path:** This variable is a string which consists of the file path of the .csv file containing columns for name, accession, and FASTA, in addition to PP, PPY, PPxY, and LPxY for the entire interactome. The file need not exist before the start of the analysis, but it should end in .csv.
  - **summary_path:** This variable is a string which consists of the file path to a .txt file that will be created containing written information about the breakdown of motifs present in the interactome. The file need not exist before the start of the analysis, but it should end in .txt.
  - **weblogo_path**: This variable is a string which consists of the file path to a .txt file with sequences 10 amino acids upstream and downstream the PPxY and LPxY motif. The file need not exist before the start of the analysis, but it should end in .txt.
  - **accessions_path:** This variable is a string which consists of the file path to a .txt file containing a list of UniProt accessions for all interactors of the protein of interest (for use in PANTHER). The file need not exist before the start of the analysis, but it should end in .txt.
