# Supplementary material for: Predicting PY motif-mediated protein-protein interactions in the Nedd4 family of ubiquitin ligases
Source: PLoS One. 2021 Oct 12;16(10):e0258315. doi: 10.1371/journal.pone.0258315 (PMC8509885; doi:10.1371/journal.pone.0258315)
Supplement: Attachment — Submitted filename: Response to Reviewers.docx [file pone.0258315.s014.docx]

**Response to Reviewers**

**Reviewer 1:**

Reviewer 1 commented on the claim that “PPxY motifs are more likely to occur in proline rich regions,” noting that it is not clear if proline-rich regions are enriched for PPxY motifs compared to what would be expected by chance. To address this, we conducted the suggested analysis wherein proteins with reported compositional bias for proline residues were retrieved from UniProt and analyzed with PxYFinder. This analysis revealed that PY motifs occur with greater prevalence in the Nedd4 family interactomes than they do in the proline rich regions queried. A discussion of this analysis is provided on Page 7.

Reviewer 1 further provided thoughtful feedback about the occurrence of end-effects in IUPred2A and noted a possible issue with our implementation of the tool to analyze extracted PY sequences instead of full interactor sequences. To address this issue, we first re-analyzed the full Nedd4-1 interactome with complete protein Fasta sequences and compared this to the results garnered with extracted PY sequences. We observed that use of the extracted sequences did in fact result in a slightly higher estimation of disorder relative to full sequences, but nonetheless the overall conclusion of the analysis was the same (i.e. PPxY occur in more disordered regions than LPxY). The IUPred2A data has been updated to include the data from analysis of complete protein Fasta sequences, and further analyses were included on the PY-motif containing interactors of SMURF1 and WWP1 as part of the response to Reviewer 2 concerns addressed above. Figure 5 and methods have been also updated accordingly.

On a related note, we also conducted Reviewer 1’s recommended control study where we computationally flipped PPxY motifs for LPxY motifs and vice versa. In this analysis, provided in a new supplemental figure (Figure S3), we determined that swapping P for L decreased the predicted disorder and L for P increased the predicted disorder, indicating that the first P residue in the motif contributes greatly to the overall estimation of disorder in the region. This trend was retained across the ligase interactomes studied. In addition to the supplemental figure, a brief discussion of this experiment was added on Page 9. This was a straight-forward but informative control experiment, and we greatly appreciate the suggestion!

Finally, Reviewer 1 recommended that a brief discussion of hydrophobicity of L vs P were added on Page 11. We added a brief discussion of this as requested.

**Reviewer 2:**

Reviewer 2 noted that the interacting proteins were retrieved from BioGrid with *Homo sapiens* as an organismal filter and detailed several of the interaction identification methods that are compiled in the database. Based on this, the reviewer requested that we discuss the reliability of these data and any bias that may occur in the datasets based on the types of methods employed. To this end, we provided a short discussion of methods used to experimentally identify interacting proteins in the Results section on Page 4.

Reviewer 2 requested that we incorporate a discussion of the possibility that proteins may contain a PY motif but not interact with Nedd4 family proteins. A short discussion of this idea was added to the Discussion section on Page 16.

The reviewer further requested that we expand the discussion of difference among the Nedd4 family substrates. To address these concerns, several revisions were made including:

1. The UpSet plot analysis in Figure 2 was complemented with a functional annotation of known interactors of each ligase using Gene Ontology terms via the PANTHER database. This additional analysis furthered the idea that each ligase is functionally distinct. Specifically, it revealed that, while there are similar trends in the overall biological processes affected by each ligase, there are distinct patterns in protein class amongst the interactomes across the Nedd4 family. This data was added to Figure 2 as Figure 2B.
2. In the initial submission, the subsequent bioinformatic analyses focused on Nedd4-1 only. To account for the lack of overlap across the family (and therefore the lack of generalizability), we chose to expand these analyses to also include WWP2 and SMURF1. As we have now explained on page 7, the analyses now include these three ligases “as these members of the Nedd4 family have the largest annotated interactome datasets (**Figure 2A**) and show distinct patterns in the types of protein classes with which they interact (**Figure 2B**).” We feel that this addition allows for analyses across ligases with distinct specificities to see if our findings are more generalizable. However, by limiting to the largest interactome datasets, we limit potential biases that may occur with inclusion of limited datasets such as HECW1, SMURF2, WWP1, which include fewer than 100 interactors. Interestingly, the sequence and structural context (as determined via the included analyses of RSA, disorder, etc.) show consistent trends across the ligases studied. We feel that the conservation of the trends across Nedd4-1, SMURF1, and WWP2 shows that the results are generalizable despite differences in substrate specificity and points to a level of regulation above that of sequence or structure context of PY motifs alone.

Additionally, the additional minor errors in the manuscript identified by both Reviewers have been corrected in this version of the manuscript as requested:

1. Clarification of the pie chart in Figure 2
2. Revision of a few sentences that were written with confusing or unclear wording
3. Correction of numbering/lettering in Figure 6 caption
4. Removal of redundant references (37 and 38 in original submission)
